# A Review on Dehydration of C(-A)-S-H and Rehydration of Dehydrated C(-A)-S-H for Recycled Cement

**DOI:** 10.3390/ma19061133

**Published:** 2026-03-14

**Authors:** Ruisong Wang, Junjie Wang

**Affiliations:** 1 Beijing No. 4 Municipal Construction Engineering Co., Ltd., Beijing 101113, China; 18601126104@163.com; 2 Department of Civil Engineering, Tsinghua University, Beijing 100084, China

**Keywords:** dehydration, rehydration, C(-A)-S-H, recycled cement, recycled concrete

## Abstract

Calcium silicate hydrate (C(-A)-S-H) and its aluminosilicate counterpart (C-A-S-H) constitute the principal binding phases in Portland cement and blended systems, governing mechanical strength and durability. This paper presents a summary of the work related to dehydration of C(-A)-S-H and rehydration of dehydrated C(-A)-S-H. Their thermal dehydration, a key process for cement recycling, induces profound multi-scale transformations: at the atomic level, it alters calcium and aluminum coordination environments and disrupts chemical bonding; at the chain-structure level, it causes depolymerization of the silicate/aluminosilicate networks; and at the microstructural level, it leads to changes in nanoscale particle morphology, aggregation state, and pore structure, creating a metastable, defect-rich, high-energy state distinct from the original C(-A)-S-H. The subsequent rehydration of this dehydrated C(-A)-S-H, which is not a simple reversal but a distinct dissolution–precipitation process, enables microstructural reconstruction and restored reactivity upon contact with water. This rehydration capacity is fundamentally exploited in thermally activated recycled cement—a novel binder concept that leverages dehydration-induced metastability for renewed strength development. Understanding these interconnected processes, influenced by factors like temperature, humidity, rate, and aluminum content, is critical for advancing sustainable cement technology, enabling the design of high-performance recycled cement and concrete, and facilitating the recycling of cementitious materials.

## 1. Introduction

Cement-based materials, predominantly concrete and mortar, are the world’s most consumed man-made substances due to their versatile properties. Their long-term performance is fundamentally governed by their key binding phase: calcium silicate hydrate (C(-A)-S-H) in ordinary Portland cement, or its aluminum-substituted form, calcium aluminosilicate hydrate (C-A-S-H), when supplementary cementitious materials are used [1]. These poorly crystalline, nanoscale phases, constituting 50–70% of the hydration products, provide cohesion, strength, and durability through their high surface area and water retention capacity.

In practical service, these materials are frequently subjected to environmental conditions that induce C(-A)-S-H dehydration, ranging from low humidity and temperature fluctuations to extreme events like fire. This dehydration is a physicochemical process involving the loss of both physically adsorbed and chemically bound water. It triggers profound multi-scale transformations: at the atomic level, through breakage or polymerization of silicate/aluminosilicate chains and changes in cation coordination; and at the microstructural level, through nanoparticle aggregation, pore coarsening, and increased crystallinity. These changes directly degrade macro-performance, with moderate dehydration causing minor strength loss and shrinkage, while severe dehydration can lead to significant strength reduction, increased permeability, and structural damage.

The thermal dehydration of C(-A)-S-H, the nanoscale binding phase of cement, is the pivotal scientific process enabling recycled cement technology. It should be noted that there are several more economical and simpler ways to recycle concrete without activation the cementitious materials than through thermal dehydration. When subjected to controlled thermal activation, C(-A)-S-H does not simply lose water; it undergoes a deliberate, multi-scale transformation from a stable hydrate to a metastable, reactive precursor. This involves the removal of both physically adsorbed and chemically bound water molecules, which triggers a cascade of structural alterations: at the atomic level, silicate and aluminosilicate chains depolymerize, and calcium coordination environments change; at the nano-scale, particles aggregate and the pore network coarsens. Crucially, this process is not pushed to complete decomposition but is carefully arrested to preserve a defect-rich, high-energy state. The resulting dehydrated C(-A)-S-H retains a “structural memory,” possessing a pronounced thermodynamic drive to recombine with water, which forms the core reactivity mechanism for a novel binder.

The rehydration of this thermally activated, dehydrated C(-A)-S-H is the engineered response that defines the functionality of recycled cement [2,3]. Upon remixing with water, the metastable phase undergoes a dissolution–precipitation sequence, reconstructing a binding gel that restores cohesion and strength [4]. Unlike conventional hydration or pozzolanic reactions, this process relies on a controlled dehydration-rehydration cycle where C(-A)-S-H acts as both the starting material and the final product. The practical performance and viability of recycled cement hinge entirely on the delicate balance between the severity of dehydration [5]—which must be sufficient to induce high reactivity—and the preservation of rehydratability. Achieving this balance allows the transformation of hardened cement paste from a construction waste into a valuable, circular resource [6,7,8], directly addressing the cement industry’s significant carbon footprint by reducing clinker demand and closing the material loop [9,10,11,12]. Thus, this review provides an in-depth exploration on the fundamental mechanisms of dehydration of C(-A)-S-H and rehydration of dehydrated C(-A)-S-H.

## 2. Fundamental Structure of C(-A)-S-H

### 2.1. Atomic Structure of C(-A)-S-H

Calcium Silicate Hydrate (C(-A)-S-H) is most accurately described as a nanocrystalline, defective analogue of the natural mineral tobermorite. Its atomic structure consists of layered calcium oxide (CaO) sheets, sandwiched between parallel chains of silicate tetrahedra (SiO_4_). These silicate chains predominantly adopt a dreierketten arrangement, characterized by a repeating pattern of paired tetrahedra (Q^2^ sites) connected by a bridging tetrahedron. A key structural variable is the Ca/Si ratio, which directly governs the polymerization degree and defect density of these chains. A lower Ca/Si ratio (closer to 1.5) promotes longer, more polymerized chains resembling 14 Å tobermorite, whereas a higher Ca/Si ratio (approaching 2.0) results in progressively shorter chains due to the omission of bridging tetrahedra, increasing overall structural disorder.

The connectivity within these silicate chains is precisely quantified using ^29^Si Magic-Angle Spinning Nuclear Magnetic Resonance (^29^Si MAS NMR) and the Q^n^ notation, where ‘n’ denotes the number of bridging oxygen atoms per tetrahedron. In typical C(-A)-S-H, the spectrum is dominated by Q^1^ (chain-end) and Q^2^ (mid-chain) silicon sites. The average silicate chain length is a direct function of the Ca/Si ratio, decreasing from approximately 4–5 at a low Ca/Si to about 2–3 at a high Ca/Si. This depolymerization critically influences the material’s surface reactivity and binding capacity. Calcium ions (Ca^2+^) reside in the interlayer spaces between the silicate chains, forming irregular coordination polyhedra with oxygen atoms from the silicate tetrahedra, hydroxyl groups, and water molecules. The interlayer also contains water molecules, which are hydrogen-bonded to the structure and play a crucial stabilizing role.

When Supplementary Cementitious Materials (SCMs) like fly ash or slag are present, aluminum (Al^3+^) substitutes for silicon in the tetrahedral chains, forming Calcium Aluminosilicate Hydrate (C-A-S-H). This Al-for-Si substitution creates a charge imbalance, which is compensated by the uptake of additional Ca^2+^ or alkali ions (Na^+^, K^+^) into the interlayer. This charge-balancing mechanism significantly alters the stability and chemical properties of the phase compared to pure C(-A)-S-H.

The interlayer region is a dynamic space containing water molecules and charge-balancing cations. The water exists in states of varying binding energy, from loosely held to bound more strongly. It acts as a structural lubricant and facilitates ion transport. The overall structural integrity of C(-A)-S-H arises from a combination of strong covalent bonds within the silicate/aluminosilicate chains (Si–O), ionic bonds between calcium and oxygen (Ca–O), and a network of weaker but numerous hydrogen bonds involving interlayer water. This hierarchy of bonding creates a robust adaptable three-dimensional network responsible for the cohesive strength of cement paste.

### 2.2. Microstructure of C(-A)-S-H

The microstructure of C(-A)-S-H is hierarchical, including nanoscale, microscale, and mesoscale structures, which are closely related to its atomic structure and hydration conditions.

At the nanoscale (5–100 nm), C(-A)-S-H exists as colloidal particles with irregular shapes, including fibrous, flaky, reticular, and granular morphologies. The size of C(-A)-S-H colloidal particles ranges from 5–50 nm, with a specific surface area of 100–500 m^2^/g. The morphology of the particles is affected by the hydration conditions: for example, C(-A)-S-H formed by tricalcium silicate (C_3_S) hydration is mainly fibrous, while C(-A)-S-H formed by dicalcium silicate (C_2_S) hydration is flaky. For C-A-S-H, the introduction of Al leads to smaller particle size and more uniform distribution compared to C(-A)-S-H, due to the inhibition of particle aggregation by Al substitution.

At the microscale (100 nm–10 μm), C(-A)-S-H colloidal particles aggregate to form a porous network structure, which is the main binding matrix of cement-based materials. The network structure is interconnected, with pores between the particles (interparticle pores) and pores inside the particles (intraparticle pores). The pore size distribution of C(-A)-S-H ranges from a few nanometers to a few micrometers, with most pores being mesopores and micropores. The porosity of C(-A)-S-H is 30–50%, and the average pore diameter is 5–20 nm. The pore structure of C(-A)-S-H is closely related to its mechanical properties: a dense network with small pore size leads to higher strength and lower permeability, while a loose network with large pore size (average pore diameter > 20 nm) results in lower strength and higher permeability.

At the mesoscale (10 μm–100 μm), the C(-A)-S-H network structure wraps around other hydration products (e.g., calcium hydroxide (CH), ettringite (AFt), monosulfate (AFm)) and aggregates, forming a continuous matrix. The interface between C(-A)-S-H and other phases is dense, with strong bonding strength, which is crucial for the overall performance of cement-based materials. For C-A-S-H, the interface bonding strength is higher than that of C(-A)-S-H, due to the smaller particle size and more uniform distribution, which enhances the mechanical interlocking effect between C(-A)-S-H and aggregates.

The crystallinity of C(-A)-S-H is very low, which is due to the disordered arrangement of silicate/aluminosilicate chains and calcium coordination polyhedra, as well as the presence of structural defects (e.g., missing Ca^2+^ ions, broken chains). The main diffraction peaks of C(-A)-S-H in XRD are broad and weak. Compared to C(-A)-S-H, C-A-S-H has slightly higher crystallinity, due to the ordering effect of Al substitution on the tetrahedral chains.

## 3. Dehydration Mechanisms of C(-A)-S-H

The dehydration of calcium (alumino) silicate hydrate (C(-A)-S-H) under elevated temperatures proceeds through distinct stages, primarily driven by the loss of water molecules residing in different binding environments. Initially, weakly bound water—such as pore water and interlayer water—is removed at relatively low temperatures (below 100–150 °C), leading to significant shrinkage of the interlayer spacing and a reduction in overall volume, as observed via in situ XRD and TEM [1]. At higher temperatures (150–400 °C), strongly bound water and hydroxyl groups (Si-OH) are eliminated through dehydroxylation, which promotes the conversion of Si-OH to Si-O-Ca/Na bonds and induces further structural densification. In extreme heating (above 400 °C), continued dehydration accompanies phase transformations, such as the collapse of the layered structure into nanocrystalline phases like 9 Å tobermorite or the formation of disordered dicalcium silicate (like larnite), and eventually, at temperatures exceeding 800 °C, the conversion to crystalline CaSiO_3_ (wollastonite) [1]. Throughout this process, water loss is accompanied by volumetric shrinkage, increased atomic disorder, and a stiffening of the solid C(-A)-S-H grains, though the overall packing density of the gel may decrease due to pore coarsening [13,14].

### 3.1. Atomic Changes During Dehydration of C(-A)-S-H

At the atomic level, high-temperature dehydration causes severe breakage of silicate/aluminosilicate chains, significant changes in calcium/aluminum coordination environments, and even the formation of new phases.

High-temperature dehydration of C(-A)-S-H induces significant collapse of its atomic structure, initiated by the rupture of the hydrogen-bonding network as structural water is removed. This triggers severe depolymerization of the silicate (in C(-A)-S-H) or aluminosilicate (in C-A-S-H) chains. The relative content of polymerized Q^2^ units decreases, while isolated Q^0^ units increases. The mean silicate chain polymerization degree decreases, indicating most chains fragment into isolated tetrahedra. Concurrently, the loss of O-H and H-O-H vibrational peaks, signifying the removal of most interlayer and chemically bound water.

The dehydration process completely reorganizes the coordination environments of key cations [15]. For calcium, the original Ca sites vanish, replaced by a new and highly distorted, low-coordination state. This reflects the breakage of original Ca-O bonds and subsequent ionic migration [16]. In C-A-S-H, aluminum undergoes a major coordination shift, a restructuring that contributes to greater stability against dehydration compared to pure C(-A)-S-H.

The atomic-scale disintegration creates a highly reactive, defect-rich state, but beyond a critical temperature threshold, it leads to irreversible phase changes. At very high temperatures, the isolated silicate/aluminosilicate tetrahedra and calcium ions recrystallize into entirely new, stable mineral phases. C(-A)-S-H transforms into wollastonite (CaSiO_3_), while C-A-S-H forms anorthite (CaAl_2_Si_2_O_8_). This crystallization represents the point where the original nanocrystalline, hydrate structure of C(-A)-S-H is destroyed and its capacity for rehydration is lost.

### 3.2. Silicate Chain Evolution During Dehydration

**Figure 1:**
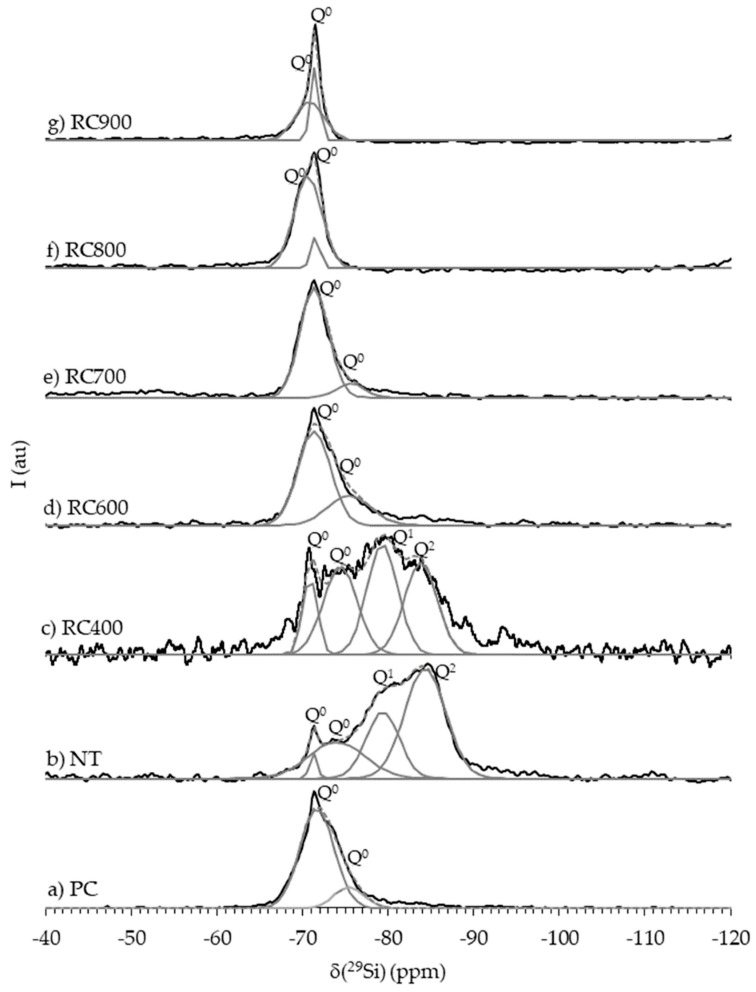
^29^Si-NMR spectra of PC (Portland cement), NT (non-treated waste cement paste) and RC (recycled cement) [18].

The dehydration of C(-A)-S-H triggers a profound and progressive depolymerization of its silicate/aluminosilicate chains. As temperature increases, the bridging tetrahedra that connect the chains are preferentially destabilized and broken [17]. This selective breakdown leads to a significant reduction in the average silicate chain length, converting longer, more polymerized structures into shorter segments dominated by Q^1^ (end-chain) and Q^2p^ (paired) units (Figure 1). This depolymerization effectively “resets” the silicate structure to a less polymerized, more reactive state of an earlier stage of hydration.

The created non-bridging oxygen atoms at the chain ends act as highly reactive dissolution sites [15]. This structural modification is central to the concept of recycled cement, as it directly governs the material’s renewed chemical reactivity. The density of these sites dictates the kinetics of the subsequent rehydration process, accelerating dissolution–precipitation reactions and thereby controlling the early-age strength development of the recycled binder. Thus, the extent of silicate chain depolymerization is a critical atomic-scale parameter that links the thermal activation process to the efficiency of structural recovery and performance regeneration.

### 3.3. Atomic Rearrangement and Calcium Coordination

During dehydration, calcium ions (Ca^2+^) undergo a critical coordination change from their original hydrated, distorted polyhedral environments to more compact, lower-coordination states [17]. This transformation is driven by the loss of coordinated water molecules, which strengthens the remaining Ca-O bonds but significantly reduces structural flexibility [19]. Concurrently, the collapse of the interlayer spacing triggers a redistribution of calcium atoms, with spectroscopic studies indicating migration towards the surfaces of the depolymerizing silicate chains [20]. These atomic-scale rearrangements—reduced coordination, stronger bonding, and ionic migration—are essential for rebalancing charge following silicate chain breakdown and directly lead to the densification of the overall microstructure [21].

The atomic rearrangements do not produce a uniform, stable structure; instead, they generate a high density of structural defects, including under-coordinated calcium sites and broken silicate bridges. These defects represent stored internal energy, transforming the dehydrated C(-A)-S-H into a chemically activated, metastable solid. The coexistence of these highly soluble, unstable calcium sites alongside more stable regions is a key novelty, explaining the rapid heat release and accelerated dissolution observed during rehydration. This defect-driven reactivity is fundamental to recycled cement, as the release of stored energy upon rewetting drives the rapid precipitation of a new binding phase, often with a distinct chemical signature.

### 3.4. Microstructural Changes During Dehydration

**Figure 2:**
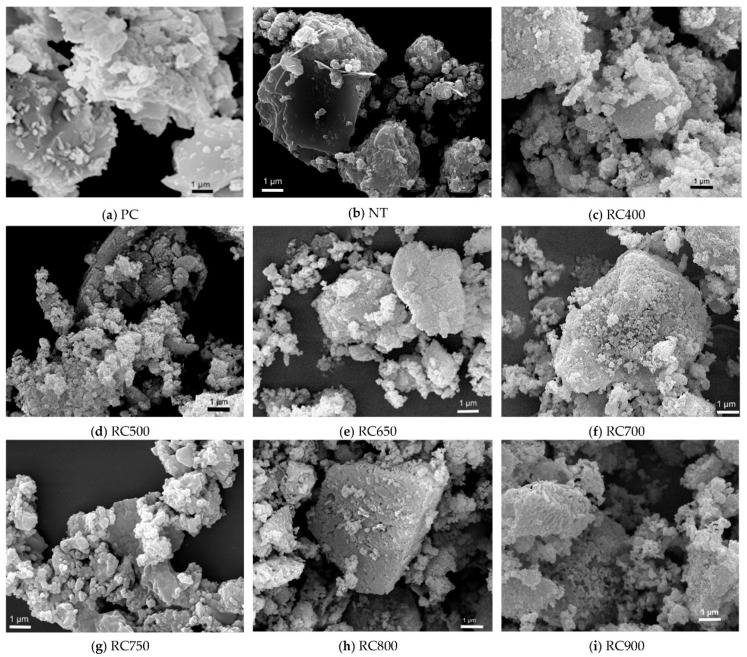
Morphologies of PC (Portland cement), NT (non-treated waste cement paste) and RC (recycled cement) [18].

At the microstructural scale, dehydration of C(-A)-S-H leads to a radical destruction of its original nano colloidal network [22]. Characteristic foil-like, fibrous, or flaky morphology of the hydrated gel collapses into granular, amorphous states [23]. This is accompanied by severe particle coarsening, where primary particle sizes increase dramatically, forming dense agglomerates up to several micrometers in size (Figure 2) [24,25]. Concurrently, the specific surface area decreases significantly.

The loss of interlayer and chemically bound water triggers the collapse of fine gel pores and the coalescence of smaller pores into larger ones [26,27]. The volume of macropores increases substantially, while the content of smaller mesopores and micropores decreases [28]. This results in an overall increase in total porosity and a coarsening of the average pore diameter [29,30]. The combined effect of severe shrinkage and particle aggregation renders the once-continuous binding network discontinuous and loose, often introducing a system of microcracks that further degrade structural integrity [31,32,33].

Notably, the microstructural degradation is less severe in calcium aluminosilicate hydrate (C-A-S-H) compared to its aluminum-free counterpart (C(-A)-S-H). Due to the stabilizing effect of Al^3+^ substitution in the silicate chains, C-A-S-H exhibits relatively less particle aggregation, a smaller reduction in specific surface area, and a more moderate increase in pore size. Its pore structure remains more dominated by mesopores, and the resulting network retains greater continuity with fewer and narrower microcracks [34,35]. This inherent stability contributes to C-A-S-H’s generally higher resistance to complete structural collapse during dehydration.

These comprehensive microstructural alterations have dual and opposing consequences for material performance. On one hand, they are directly detrimental to mechanical properties in the dry state, causing significant strength loss due to increased porosity, microcracking, and the loss of cohesive surface area [36]. On the other hand, this very transformation is essential for the reactivity of recycled cement. The newly created and highly accessible internal surfaces, along with the interconnected pore and crack network, provide abundant pathways for water ingress and numerous nucleation sites for the precipitation of new binding phases during rehydration, thereby enabling effective reactivation.

## 4. Rehydration Mechanisms of Dehydrated C(-A)-S-H

Rehydration of dehydrated C(-A)-S-H involves the reabsorption of water molecules into the altered structure, though the extent of recovery depends on the severity of prior dehydration. When dehydrated at moderate temperatures (below 400 °C), the material can partially rehydrate upon cooling and exposure to moisture, with water re-entering the interlayer spaces and pores, leading to some recovery of interlayer spacing and gel porosity. However, rehydration is often incomplete because dehydroxylation and the associated formation of new Si-O-Ca/Na bonds reduce the availability of hydrophilic sites, while structural collapse and phase transformations (e.g., to tobermorite or CaSiO_3_) create less accessible, more crystalline regions. In cases of high-temperature dehydration (above 800 °C), where irreversible phase changes and sintering occur, rehydration capacity is significantly diminished; the resulting crystalline phases (e.g., wollastonite) and densified morphologies exhibit limited ability to reabsorb water, leading to permanent microstructural changes and reduced re-cementing potential.

### 4.1. Atomic and Chain Reconstruction During Rehydration

**Figure 3:**
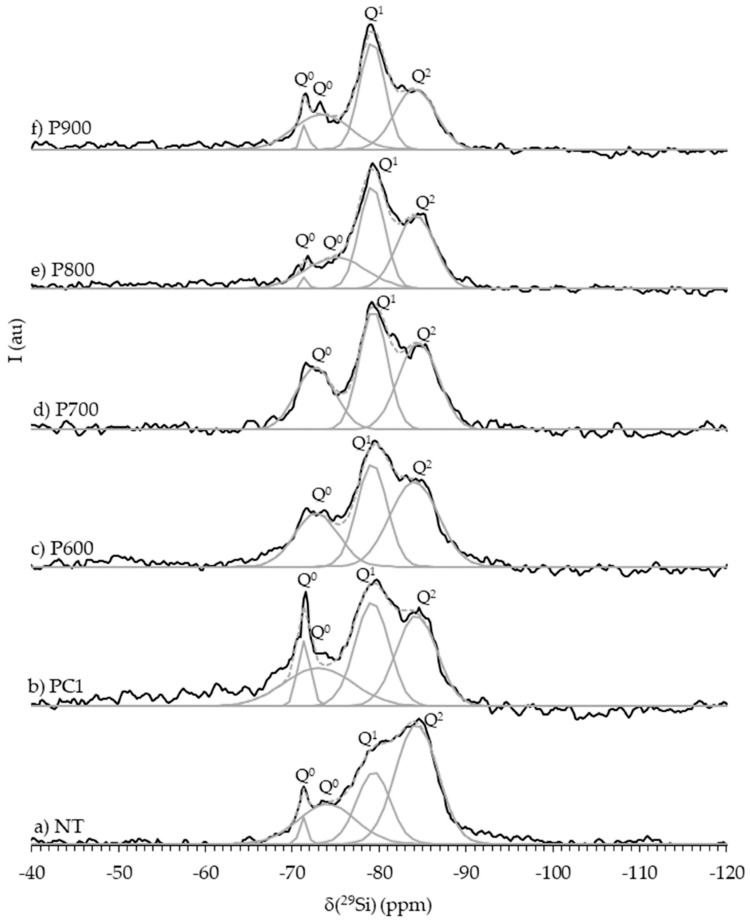
^29^Si-NMR spectra of NT (non-treated waste cement paste) at 90 days, PC1 (Portland cement) paste and RC pastes at 28 d of hydration [18].

Upon contacting with water, rehydration of dehydrated C(-A)-S-H initiates a process of silicate chain reconstruction, primarily driven by the re-incorporation of water and the subsequent dissolution and repolymerization of silicate species. The relative contents of Q^1^ (end-chain) and Q^2^ (mid-chain) units gradually return to their original levels over time (Figure 3). Concurrently, the population of isolated Q^0^ units drastically decreases. This shift indicates the reformation of silicate chains, restoring the average degree of polymerization to its original range. The chemical shifts of these silicon sites also return to their pre-dehydration values, signifying the recovery of the local electronic environment around the silicon atoms. The recovery extends to the coordination spheres of key cations. For calcium, the distorted, low-coordination state formed during dehydration disappears upon rehydration. The original Ca sites are re-established, and their intensity ratio returns [37]. In C-A-S-H, aluminum also reverts to its initial coordination state, with majority of Al^3+^ shifts back from octahedral to tetrahedral coordination within the aluminosilicate chains. The re-entry of water molecules into the layered structure is fundamental to the structural restoration. The recovery of the hydrogen-bonding network happens. The Si-O stretching vibration peak also shifts back to its original state, confirming the restoration of the silicate chain connectivity [37].

### 4.2. Microstructural Recovery and Mechanical Implications

**Figure 4:**
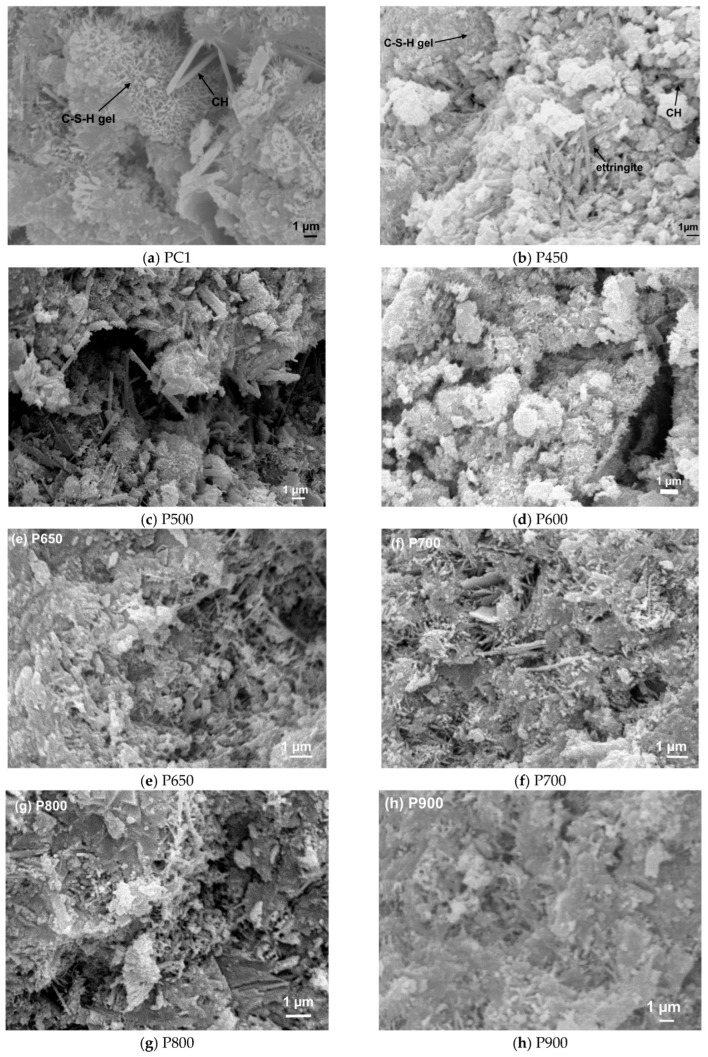
Microstructure of PC (Portland cement) paste and recycled cement pastes at 28d of hydration [18].

During rehydration, the dehydrated and aggregated microstructure of C(-A)-S-H undergoes significant restoration [38]. The large, dense agglomerates formed during dehydration gradually re-disperse, and the original fibrous or foil-like colloidal morphology is largely recovered (Figure 4), with primary particle sizes returning to the nanometer range [39]. The rehydration process was conducted under room temperature with harden Portland cement paste treated under different temperatures. Correspondingly, the specific surface area increases significantly, recovering most of its original state potentially. Total porosity decreases and the average pore diameter shifts back towards smaller sizes, restoring a pore size distribution once again dominated by mesopores and micropores [40]. Furthermore, the microcracks generated from dehydration shrinkage are partially filled and bridged by newly precipitated gel, contributing to a more continuous network. While the final restored atomic structure is remarkably complete for material dehydrated at moderate temperatures, the kinetics of recovery differ between C(-A)-S-H and C-A-S-H. Due to factors like a more uniform initial particle distribution and the stabilizing role of aluminum, C-A-S-H often exhibits a slightly faster rehydration rate, achieving most of atomic-level recovery fast. This process results not in a simple reversal to an aged structure, but in the re-formation of a binding phase that is highly reactive, which is crucial for the development of strength in recycled cement applications.

This microstructural recovery is not a simple solid-state reabsorption of water but is predominantly driven by a dissolution–precipitation mechanism. The dehydrated material, rich in under-coordinated calcium sites and non-bridging oxygen atoms, exhibits high solubility upon water exposure. Rapid dissolution of these metastable phases elevates the solution pH and increases the concentration of calcium and silicate ions, which then reprecipitate as a new, secondary C(-A)-S-H gel. This newly formed gel fills pores and cracks, leading to the observed microstructural refinement. The process is inherently faster for C-A-S-H than for C(-A)-S-H, likely due to Al-substitution promoting a more uniform and reactive initial state after dehydration.

The reformation of the binding gel directly translates to the recovery of mechanical strength, particularly early-age strength, which is governed by the rapid nucleation and precipitation of this secondary C(-A)-S-H [41]. However, the recovery is often partial. The newly formed gel tends to have a distinct atomic structure—often featuring shorter silicate chains and a different Ca/Si ratio—and does not perfectly replicate the original, mature hydrate. Consequently, while porosity is refined, a coarser residual pore network and intrinsic structural defects typically persist. This results in a final material that, despite regaining substantial load-bearing capacity, frequently exhibits reduced ultimate strength and potentially compromised long-term durability (e.g., increased susceptibility to carbonation and chloride ingress) compared to the original cement paste. Therefore, the microstructural and mechanical recovery of dehydrated C(-A)-S-H underscores the central balance in recycled cement technology. The thermal activation (dehydration) must be severe enough to generate the high reactivity needed for effective rehydration and strength development, yet controlled enough to avoid creating excessive, irreversible damage that limits final performance. Optimizing this balance is critical to maximizing the efficiency of recycled cement binders, transforming thermally activated paste from a degraded material into a functionally reactive resource.

## 5. Implications for Recycled Cement Technology

**Figure 5:**
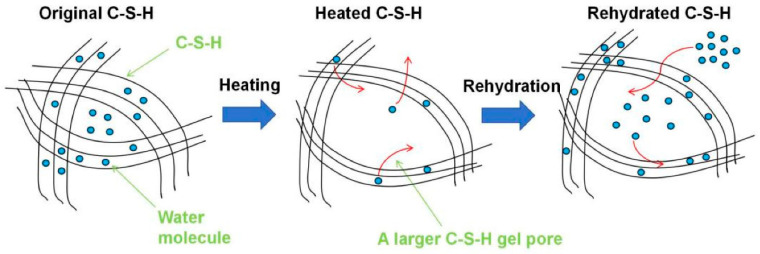
Simplified sketch on dehydration and rehydration of C-S-H for recycled cement [54].

Understanding dehydration and rehydration mechanisms enables optimization of recycled cement production (Figure 5). Control of thermal treatment temperature and duration can enhance reactivity while minimizing irreversible structural collapse [42]. The atomic-scale understanding of C(-A)-S-H dehydration and rehydration provides a direct scientific foundation for optimizing recycled cement production. The central challenge lies in precisely controlling thermal activation parameters [43]—temperature, heating rate, and residence time—to maximize the creation of a metastable, reactive state [44] while minimizing irreversible structural collapse and crystallization (e.g., to wollastonite). Lower temperatures with sufficient duration may better preserve some silicate connectivity for long-term durability, whereas slightly higher temperatures can generate greater defect density for higher early reactivity [45]. This process optimization aims not at complete decomposition [46,47] but at achieving a desired state of reversible dehydration, transforming aged C(-A)-S-H into a chemically activated precursor [48]. Furthermore, blending the recycled cement with supplementary cementitious materials (SCMs) [49,50,51,52] can introduce additional silicates and aluminates, improving the chemistry of the pore solution during rehydration and promoting the formation of a more robust and durable rehydrated gel [53].

The rehydration of thermally activated C(-A)-S-H is fundamentally distinct from conventional clinker hydration, a fact with significant implications for technology development [55,56]. Unlike clinker hydration, which involves the dissolution of crystalline anhydrous phases, recycled cement relies on the dissolution–precipitation of a metastable, amorphous dehydrated gel. This results in different reaction kinetics, with rapid early heat release and strength gain from fast precipitation, but potentially different long-term microstructural development. Consequently, conventional performance models for ordinary Portland cement, such as prediction for strength and durability at early ages, may not be directly applicable. Recognizing this distinction is essential; the performance of recycled cement binders must be evaluated based on their own mechanistic principles, balancing the high early reactivity afforded by atomic-scale defects against the need for long-term microstructural refinement and durability. This knowledge guides not only process control but also the development of appropriate application standards for this sustainable material.

## 6. Future Research Directions

Future studies could focus on quantitative correlation between atomic-scale changes and macroscopic performance. Advanced in situ characterization techniques can provide real-time insights into structural evolution. Development of predictive models integrating thermodynamics and kinetics [57] will aid the design of high-performance recycled cement materials. A primary future research direction lies in establishing robust, quantitative correlations between the atomic-scale structural changes during C(-A)-S-H dehydration/rehydration and the resulting macroscopic performance of recycled cement. Key gaps exist in precisely linking metrics like defect density and silicate depolymerization degree to engineering properties such as strength development, shrinkage, and long-term durability. Closing these gaps necessitates the development and application of advanced in situ characterization techniques (e.g., high-temperature NMR, synchrotron-based XRD) to capture real-time structural evolution, coupled with multi-scale predictive models. Integrating thermodynamics, kinetics, and machine learning could be applied to move from empirical optimization to the rational design of high-performance recycled cement formulations [58], effectively translating atomic insights into industrial practice [59].

Future work could also focus the fundamental science governing recycled cement, recognizing its distinct mechanism based on microstructural transformation rather than the chemical composition or phase transformation central to clinker or alkali-activated systems. This involves a detailed investigation into the structural reversibility—understanding how the permanent alterations in chain topology and calcium heterogeneity influence both the material’s reactivity and durability. Consequently, this distinct paradigm necessitates the development of new performance metrics, activation protocols, and long-term durability models specifically tailored for recycled cement, establishing it as a sustainable binder material.

## 7. Conclusions

Dehydration and rehydration of C(-A)-S-H involve complex structural transformations spanning atomic to microstructural scales. The following conclusions can be drawn from this review work:

(1) The dehydration and rehydration of C(-A)-S-H constitute a complex, partially irreversible cycle of structural transformation that is central to the science of recycled cement. Dehydration, through controlled thermal activation, deliberately induces silicate chain depolymerization, rearrangement of calcium coordination, and collapse of the gel microstructure, generating a metastable, defect-rich state. Subsequently, rehydration proceeds not as a simple reversal but via a dissolution–precipitation pathway, where these atomic-scale defects drive rapid dissolution and reprecipitation of a secondary, structurally distinct C(-A)-S-H gel. This fundamental mechanism, exploiting structural metastability rather than the chemical composition of traditional clinkers, could indicates recycled cement as a novel class of binder with a unique reactivity profile.
(2) The performance of recycled cement is a direct manifestation of these atomic-scale processes. The defect density created during dehydration, including non-bridging oxygen sites and under-coordinated calcium, directly dictates the kinetics of rehydration and the early-age strength development. The rapid precipitation of new C(-A)-S-H effectively bridges particles, translating nanoscale reactivity into macroscopic binding capacity. However, this very defect-rich nature also implies that the rehydrated structure is altered, with incomplete recovery of original chain lengths and calcium distribution, which links the initial high reactivity to long-term performance considerations.
(3) This understanding reveals a critical dualism. While the intentionally created defects are the source of high reactivity, they also increase the material’s vulnerability to environmental attacks, such as carbonation and ion leaching. However, this relationship also presents opportunities; for instance, controlled carbonation could potentially stabilize the rehydrated structure while contributing to CO_2_ sequestration. Thus, the central challenge and focus for process optimization lie in striking a possible balance: maximizing reversible dehydration to achieve sufficient reactivity for strength development, while minimizing irreversible damage that compromises long-term durability.
(4) The possible engineering application of recycled cement lies in harnessing controlled structural transformations of C(-A)-S-H across atomic, chain, and microstructural scales. This provides a robust framework that moves the technology beyond empirical formulation toward rational design, which could indicate that recycled cement is not merely a derivative of Portland cement but represents a distinct paradigm in binder technology. Its potential advancement and industrial implementation will depend on future research that quantifies the microstructure–performance relationship, develops tailored property models, and establishes appropriate performance metrics for this sustainable, low-carbon material.
(5) Limitations and controversies: This review cannot cover all the aspects of the dehydration and rehydration of C(-A)-S-H. The above statements are made under several hypotheses that the materials are in high purity and the test conditions are under normal room conditions. There are many limitations on this work, the performance of the dehydration and rehydration of C(-A)-S-H largely depend on the atomic composition of the C(-A)-S-H, temperature range, atmosphere, duration of the treatment method and measurement precisions.

## Data Availability

No new data were created or analyzed in this study. Data sharing is not applicable to this article.

## Abbreviations

PC	Portland Cement
NT	Non-treated
RC	Recycled Cement
SCMs	Supplementary Cementitious Materials
MAS	Magic Angle Spinning
NMR	Nuclear Magnetic Resonance
